# Microwave-Assisted Synthesis of Ge/GeO_2_-Reduced Graphene Oxide Nanocomposite with Enhanced Discharge Capacity for Lithium-Ion Batteries

**DOI:** 10.3390/nano11020319

**Published:** 2021-01-27

**Authors:** Ji-Hye Koo, Seung-Min Paek

**Affiliations:** Department of Chemistry, Kyungpook National University, Daegu 41566, Korea; guzza95@naver.com

**Keywords:** germanium oxide, graphene composite, anode material, lithium-ion battery

## Abstract

Germanium/germanium oxide nanoparticles with theoretically high discharge capacities of 1624 and 2152 mAh/g have attracted significant research interest for their potential application as anode materials in Li-ion batteries. However, these materials exhibit poor long-term performance due to the large volume change of 370% during charge/discharge cycles. In the present study, to overcome this shortcoming, a Ge/GeO_2_/graphene composite material was synthesized. Ge/GeO_2_ nanoparticles were trapped between matrices of graphene nanosheets to offset the volume expansion effect. Transmission electron microscopy images revealed that the Ge/GeO_2_ nanoparticles were distributed on the graphene nanosheets. Discharge/charge experiments were performed to evaluate the Li storage properties of the samples. The discharge capacity of the bare Ge/GeO_2_ nanoparticles in the first discharge cycle was considerably large; however, the value decreased rapidly with successive cycles. Conversely, the present Ge/GeO_2_/graphene composite exhibited superior cycling stability.

## 1. Introduction

Recently, with the increase in the demand for portable electronic devices and electric automobiles, the interest in energy storage systems has also grown. In particular, the research on next-generation electrode materials for Li secondary battery systems is actively progressing. Currently, graphite, which has a low specific capacity of 372 mAh/g, is commercially and widely used as an anode material for Li-ion batteries. To overcome the intrinsic limitation of the graphite electrode with low energy density, metal oxides and group-IV elements (Si, Ge, Sn, etc.) have been attracting attention as potential anode materials with excellent theoretical capacities and low working voltages [1,2,3,4]. In particular, Ge, our element of choice, holds promise because of its high theoretical capacity (1624 mAh/g) [5]. In addition, compared to Si, it has 400 times faster ion diffusivity and 104 times higher electrical conductivity [6,7,8,9,10].

Ge + 4.4Li ↔ Li_4.4_Ge (1)

GeO_2_ + 4Li → Ge + 2Li_2_O (2)

Ge generally stores Li ions through an alloying mechanism (1) [11,12,13,14], which yields Li_22_Ge_5_ in a fully charged state. Meanwhile, GeO_2_ undergoes conversion (2) before generating the Li–Ge alloy [15,16,17,18]. However, as the alloying-dealloying reaction proceeds, the conversion of GeO_2_ is irreversible, due to the formation of a Li^+^ inactive phase (LiO_2_) that could result in a decrease in the Coulombic efficiency (CE) [19]. This decreases the maximum expression of the theoretical capacity of GeO_2_ (2152 mAh/g) by half. Despite this limitation, its theoretical capacity remains high. On the other hand, the intrinsic drawback of Ge anode is that it generally undergoes a large volume expansion of 370% during the lithiation and delithiation process; this expansion has a tremendous impact on the stability of its electrodes. This large volume variation induces a crack and the consequent pulverization of the electrode in which the active material is physically separated from the current collector; this causes a severe decrease in the capacity of the Ge electrode for battery applications. To overcome such a demerit of Ge-containing anodes, a number of research attempts have been conducted by fabricating composites between carbonaceous materials and Ge-containing materials. For example, mesoporous Ge/GeO_2_/carbon composite with increase discharge capacity was prepared via self-assembly [20]. Recently, Ge/GeO_2_/carbon composite, prepared by carbon coating, also showed increased reversibility upon successive charge/discharge cycles [21]. The developed nanostructured pores in these Ge/GeO_2_/carbon composites could be utilized as buffered spaces to minimize volume expansion during charge/discharge, leading to the superior electrochemical lithium storage properties. In this regard, reduced graphene oxide (RGO) can be a promising alternative for preparing nanostructured composites with Ge/GeO_2_, since electrochemical and structural properties of RGO could be effectively controlled by choosing proper synthesis methods [22,23,24].

**Scheme 1:**
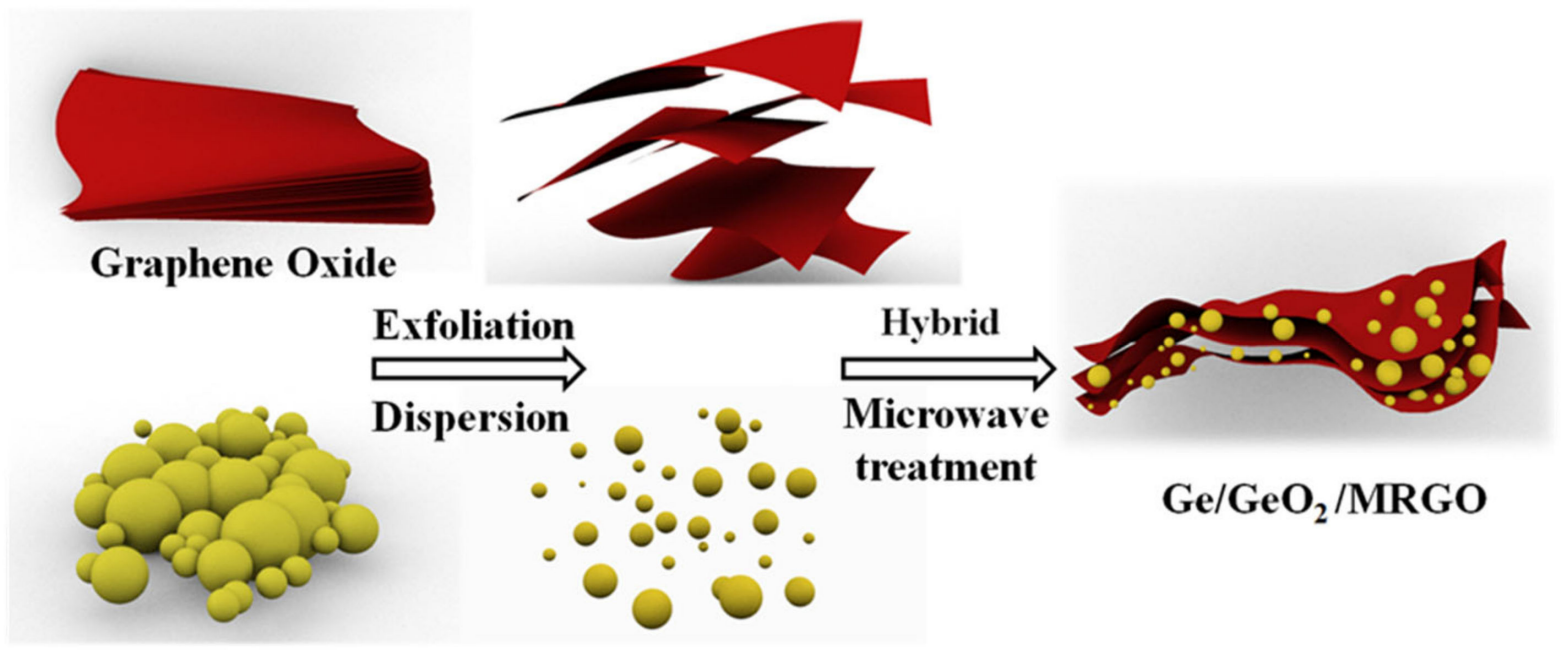
Pictorial illustration of the synthesis process.

In this contribution, we propose a new model compound, which is a hybrid of Ge/GeO_2_ and RGO (Scheme 1). This composite material was synthesized by reassembling fully exfoliated graphene oxide (GO) nanosheets in the presence of Ge/GeO_2_ nanoparticles (NPs) and subsequent treating microwave. Microwave irradiation could produce an oscillating field which induces both GO and the adsorbed water to absorb high energy within short irradiation time. Therefore, GO can be effectively reduced into RGO by using microwave treatment. According to our previous publication on microwave-treated reduced graphene oxide (MRGO) with porous structure showed highly enhanced electrochemical energy storage properties including high electrical conductivity and a short ion diffusion path. Also, the empty spaces with a corrugated structure, generated by the removal of oxygen-containing functional groups on the surface of GO through microwave treatment, can accelerate the buffering action of the Ge/GeO_2_ NPs during the lithiation/delithiation process. In addition, this double-protected structure keeps in proximity the lithium oxides and Ge that were produced during the GeO_2_ conversion, which is beneficial for the oxidation kinetics of Ge. Furthermore, the MRGO, as highly conductive additives, enhances the electron transport and ion diffusion, which compensates for the low electrical conductivity and diffusivity of Ge NPs [25,26]. Our key strategy of this research is to incorporate Ge/GeO_2_ nanoparticles into the matrices of MRGO. The role of Ge/GeO_2_ NPs in the composite is to increase energy density of the product. On the other hand, GO plays a role in the enhancement of electrical and ionic conductivity after GO was converted to MRGO by microwave treatment. Through these strategies, it was clearly shown that the present Ge/GeO_2_/MRGO had superior electrochemical performance in terms of the cycling performances, capacity retention, and rate capabilities, compared to those of the bare Ge/GeO_2_ NP anode [27,28,29,30,31,32,33,34,35].

## 2. Experimental Section

### 2.1. Sample Preparation

GO was synthesized by a modified Hummer’s method [36]. Briefly, 1 g of bulk graphite and 0.75 g of sodium nitrate were dispersed in 75 mL of concentrated H_2_SO_4_. Thereafter, 4.5 g of potassium permanganate was slowly added over 1 h while stirring in an ice bath. The colloidal suspension was continuously stirred for five days, followed by washing with 16 mL of H_2_O_2_, 16 mL of H_2_SO_4_, and 968 mL of distilled water (dispersing solvent). Additional washing was conducted using a 1:1 mixture of distilled water and ethanol, followed by drying in ambient condition to yield a brown powder of GO.

Scheme 1 shows the synthesis route of Ge/GeO_2_/MRGO. The commercially available Ge/GeO_2_ NPs (RNDKOREA, 99.9+% purity, 35 nm) were used as received. Briefly, 70 mL of distilled water was added to 0.7 g of dried GO in a flask. This aqueous suspension of GO was dispersed by treating sonication for 10 min (POWERSONIC410, 400 W). In another flask, 0.3 g of the Ge/GeO_2_ NPs was added and dispersed in a solution of 15 mL of distilled water and 15 mL of acetone under sonication for 10 min. Subsequently, the above colloidal suspensions of GO and Ge/GeO_2_ NPs were mixed and stirred for 3 h in a round-bottomed flask. The reactant was allowed to settle for 5 min, and then, gathered by centrifugation at 15,000 rpm, to yield a hybrid material of GO nanosheets and Ge/GeO_2_ NPs (Ge/GeO_2_/GO). This as-prepared product was dried at room temperature for five days and subsequently irradiated with microwaves for 10 min to obtain the product (Ge/GeO_2_/MRGO). To prevent overheating, 2.5 min of microwave treatment was conducted 4 times, in which there was 1 min of pause before each successive microwave irradiation.

### 2.2. Characterizations

X-ray diffraction (XRD) analysis of the samples was carried out using an X-ray diffractometer (Bruker D2 phaser, λ = 1.5406 Å). The electronic and local structure was analyzed by X-ray absorption spectroscopy (XAS) at the beamline 8C (Nanoprobe XAFS) in the Pohang Accelerator Laboratory, South Korea, and all spectra were obtained in the fluorescence mode at the Ge K-edge (11,103.1 eV). XAS data analysis was conducted by a standard procedure [37]. The surface features and morphology were characterized by field-emission scanning electron microscopy (FE-SEM, Hitachi SU8220) and high-resolution transmission electron microscopy (HR-TEM, JEOL JEM-2100F), in which acceleration voltages for FE-SEM and HR-TEM measurements were 30 kV and 200 kV, respectively. The Fourier transform infrared (FT-IR) spectra of the samples were obtained (Thermo Scientific Nicolet iS5, Waltham, MA, USA) to confirm the formation and extinction of functional groups during the synthesis. Raman spectra were measured by Raman spectrometer (HORIBA XploRA Plus, a laser wavelength of 532 nm). The thermal behavior of the sample was investigated during the heating process up to 800 °C by thermogravimetric analysis (TGA instruments, STA-S1000) at a heating rate of 5 °C/min. The specific surface area and pore size distribution were determined by Brunauer–Emmett–Teller (BET) measurements (Quantachrome Autosorb-iQ and Quadrasorb SI) and Barrett–Joyner–Halenda (BJH) plots, under N_2_ gas adsorption/desorption at 77 K. The electrochemical charge/discharge performance was tested by constructing CR2032 coin cells. The electrodes were composed of a viscous slurry mixture of the active materials, conducting carbon (super P), and polyacrylic acid in a 7:2:1 weight ratio, in N-methyl-2-pyrrolidone (NMP). The slurry was cast on a Cu foil using a doctor blade, followed by drying overnight (110 °C in a vacuum oven). A half-cell was fabricated using the sample-cast Cu foil as the working anode and current collector, a Li foil as the counter metal and reference electrodes, 1 M LiPF_6_ in ethylene carbonate (EC)/diethyl carbonate (DEC) (1:1 volume ratio) as the electrolyte, and polyethylene (PE) as the separator. All the cells were assembled in an Ar-filled glove box. Electrochemical impedance spectroscopy (EIS) data were obtained by impedance analyzer (WonATech, ZiVE-SP2, Seoul, Korea) at an open-circuit voltage in the frequency range of 10^−2^–10^5^ Hz. Galvanostatic charge/discharge tests (rate capability, cycling performance, and reversible capacity) were conducted with a battery cycler (Maccor K4300) in the voltage window of 0.01–3 V, at various current densities. Cyclic voltammetry (CV) analysis was carried out using a potentiostat (WonATech, WMPG1000) in the potential range of 0.01–3 V, by varying scan rates from 0.1 to 0.8 mA/g.

## 3. Results and Discussion

### 3.1. Structural and Morphological Characteristics

**Figure 1:**
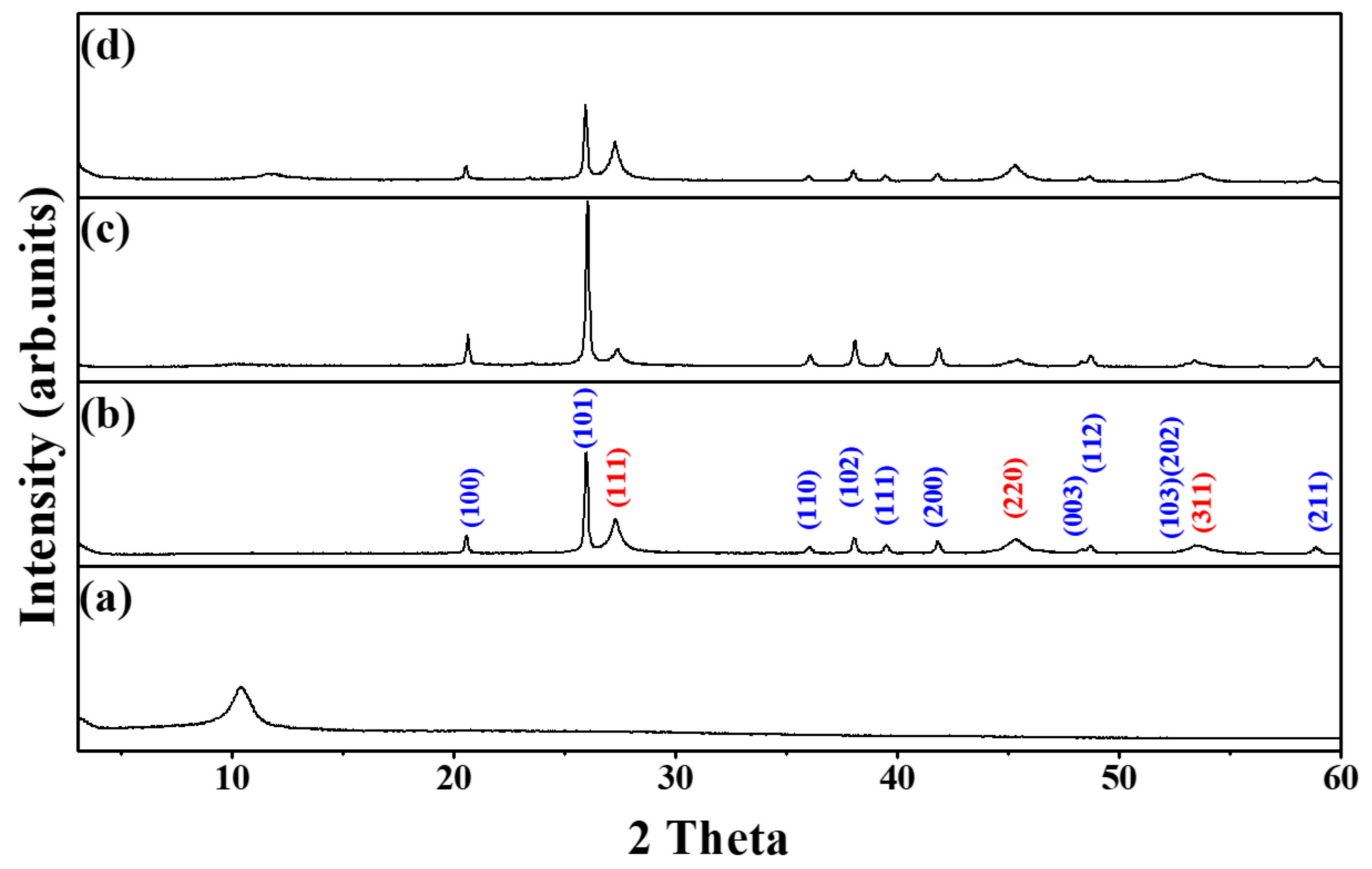
X-ray diffraction (XRD) patterns of (**a**) GO, (**b**) Ge/GeO_2_ nanoparticles (NPs), (**c**) the as-prepared composite (Ge/GeO_2_/GO), and (**d**) Ge/GeO_2_/MRGO (microwave-treated reduced graphene oxide). Blue-colored indices are from the cubic Ge, while red-colored ones are from the hexagonal GeO_2_.

To determine the crystal structure of the samples, we conducted an XRD analysis. Figure 1 shows the XRD patterns of (a) GO, (b) Ge/GeO_2_, (c) Ge/GeO_2_/GO, and (d) Ge/GeO_2_/MRGO. The peak distinguished at around 2θ = 10° corresponds to the (002) planes of GO. The basal spacing of the graphene layers with oxygen-containing functional groups, calculated via the Bragg equation, is approximately 0.84 nm, which is higher than that of conventional graphite (0.34 nm). The diffraction patterns of germanium dioxide (GeO_2_) and Ge metal can be indexed to the hexagonal structure (ICDD# 36-1436) and diamond cubic structure (ICDD# 04-0545), respectively. First, a sharp diffraction peak is observed at 2θ = 20.5°, indexed to the (101) plane of hexagonal GeO_2_. Two relatively broad peaks can be observed at 2θ = 27.3° and 2θ = 45.3°, which are indexed to the (111) and (220) reflections of the cubic Ge crystal. The Scherrer equation (D = Kλ/β cosθ) was used to calculate the particle size of the Ge metal, where D is the crystallite size, K is the Scherrer constant related to the (hkl) indexing and the morphology of the NPs, λ is the wavelength of the X-ray source (0.15406 nm), β is full width at half maximum of the peak, and θ is the XRD peak position in radians. The broad peak of Ge (220) was used to calculate the crystallite size of Ge, in which the primary particle size was determined to be 38.6 nm. Notably, compared to the pristine Ge/GeO_2_ NPs, the relative intensity of GeO_2_ to Ge in the Ge/GeO_2_/GO hybrid material was increased due to its oxygen-abundant reaction condition. Conversely, after microwave treatment, the randomly restacked GO layers were reduced. In this case, the relative intensity of GeO_2_ to Ge decreased more than that of the bare Ge/GeO_2_ NPs. This implies that microwave treatment not only removes the oxygen-containing functional group, which results in the reduction of GO, but also causes the slight destruction of crystalline GeO_2_ to amorphous GeO_2_. The relative XRD peak intensities between (111) for Ge and (101) for GeO_2_ were about 0.36 (for Ge/GeO_2_ nanoparticles) and 0.54 (for Ge/GeO_2_/MRGO), respectively. Although the amount of Ge was increased upon the microwave irradiation, both Ge and GeO_2_ still maintained their crystal structures even after microwave treatment.

**Figure 2:**
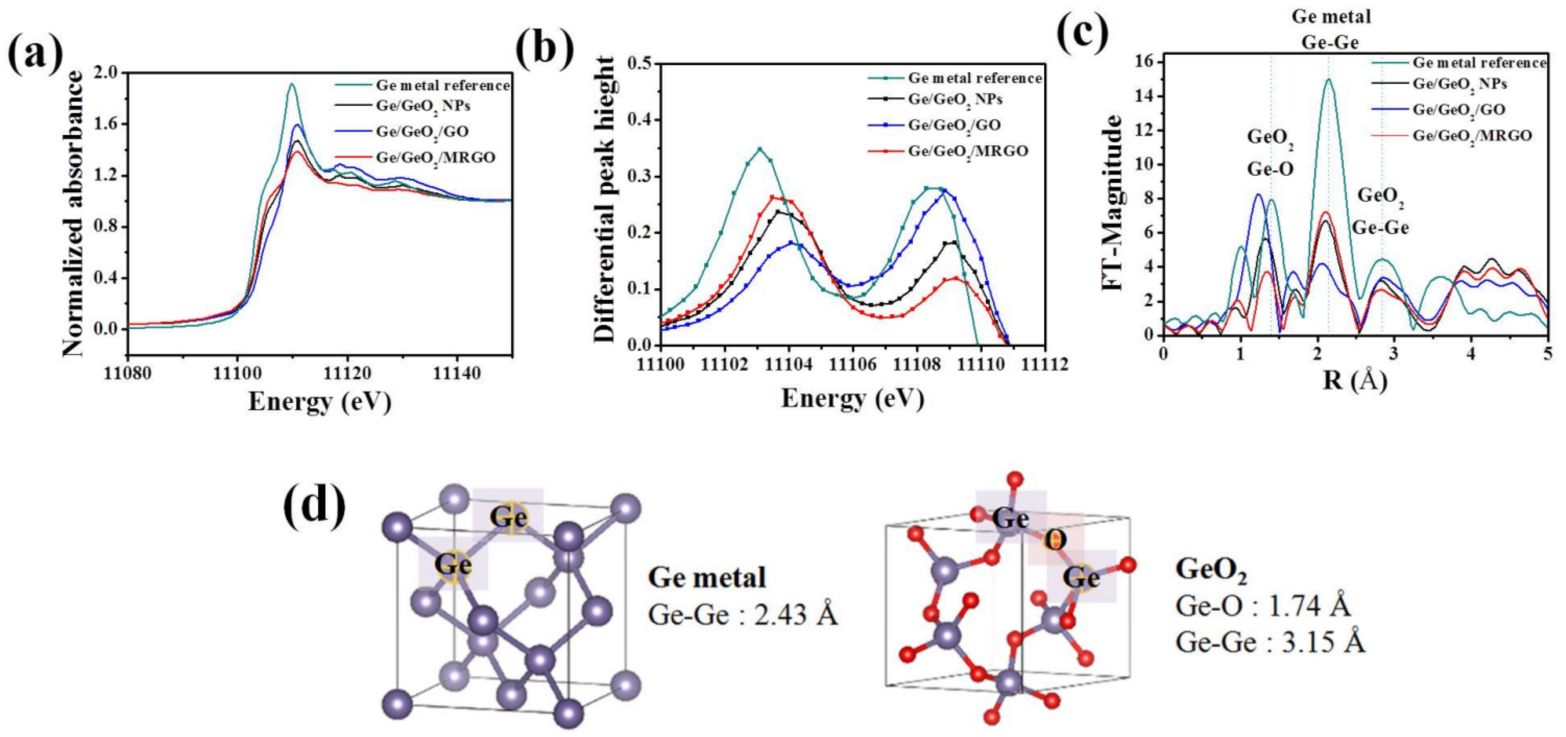
(**a**) Ge K-edge X-ray absorption near edge structure (XANES) spectra, (**b**) first derivative of the XANES spectra, (**c**) Fourier transforms (FTs) of *k^3^*-weighted EXAFS spectra of the samples, (**d**) 3D visualization of cubic Ge and hexagonal GeO_2_ crystal.

While XRD is sensitive to long-range ordering, XAS allows further studies to clarify the local structure of the compounds. Figure 2a shows the normalized X-ray absorption near edge structure (XANES) spectra detected at the Ge K-edge, and Figure 2c shows the Fourier transforms (FTs) of the extended X-ray absorption fine structure (EXAFS) spectra of the as-prepared samples before and after microwave irradiation, in comparison with those of the bare Ge/GeO_2_ NPs. As shown in Figure 2a, the edge jump for the XANES spectra of the Ge K-edge is ascribed to the electron transition from a 1s core level to an empty 4p state. This sharp increase in the absorption edge coefficient (μ(E)) occurs when the energy transmitted from the photon is higher than the threshold energy (E^0^) that excites the core electron. Based on the coexistence of metallic Ge and GeO_2_ in the samples, the sharp rising peak at around 11,108 eV corresponds to Ge^4+^ (GeO_2_), and the rising peak that appears like a shoulder in the lower energy region (around 11,103 eV) corresponds to the absorption edge of Ge^0^ (metallic Ge). By comparing these E^0^ values, we can understand the oxidation state of Ge according to the synthesis process. First, the E^0^ value of Ge/GeO_2_/GO shifted to the right side compared to the case before the reaction, and with the formation of Ge/GeO_2_/MRGO, the peak position shifted back to the left side, toward a lower energy region than that of Ge/GeO_2_/NP. In other words, during hybridization, metallic Ge is oxidized in a certain amount and subsequently reduced after microwave treatment, indicating that the reaction proceeded until the amount of metallic Ge increased higher than that in the bare Ge/GeO_2_ NPs. The E^0^ values coincide with the inflection point in the section showing the edge jump of the XANES spectra and, thus, it could be more clearly confirmed through the comparison of the maximum value in the first differential graphs. The two peaks, shown in the first differential graph in Figure 2b, correspond to the E^0^ values of Ge and GeO_2_, respectively, and the peak values of Ge/GeO_2_/MRGO are virtually identical with those of the bare Ge/GeO_2_ nanoparticles, suggesting that local and electronic structure of Ge/GeO_2_ in the composite were very similar with those of the bare Ge/GeO_2_ NPs. In the radial distribution function (RDF) plots, each shell is indexed by comparing the bond distances between the atoms obtained from hexagonal GeO_2_ and cubic Ge, displayed in Figure 2d. The distinct peaks around 1.4 Å (first shell) and 2.8 Å (third shell) correspond to the Ge–O and Ge–Ge bonds of GeO_2_, respectively, and the peak at 2.1 Å (second shell) corresponds to the Ge–Ge bonds of the Ge metal. In the XANES spectrum for Ge/GeO_2_/MRGO, it was confirmed that the intensity of the peak related to the GeO_2_ phase decreased, while that for the elemental Ge increased. From the above results, we can find that microwave treatment can be effectively used to reduce a portion of GeO_2_ into the elemental Ge.

**Figure 3:**
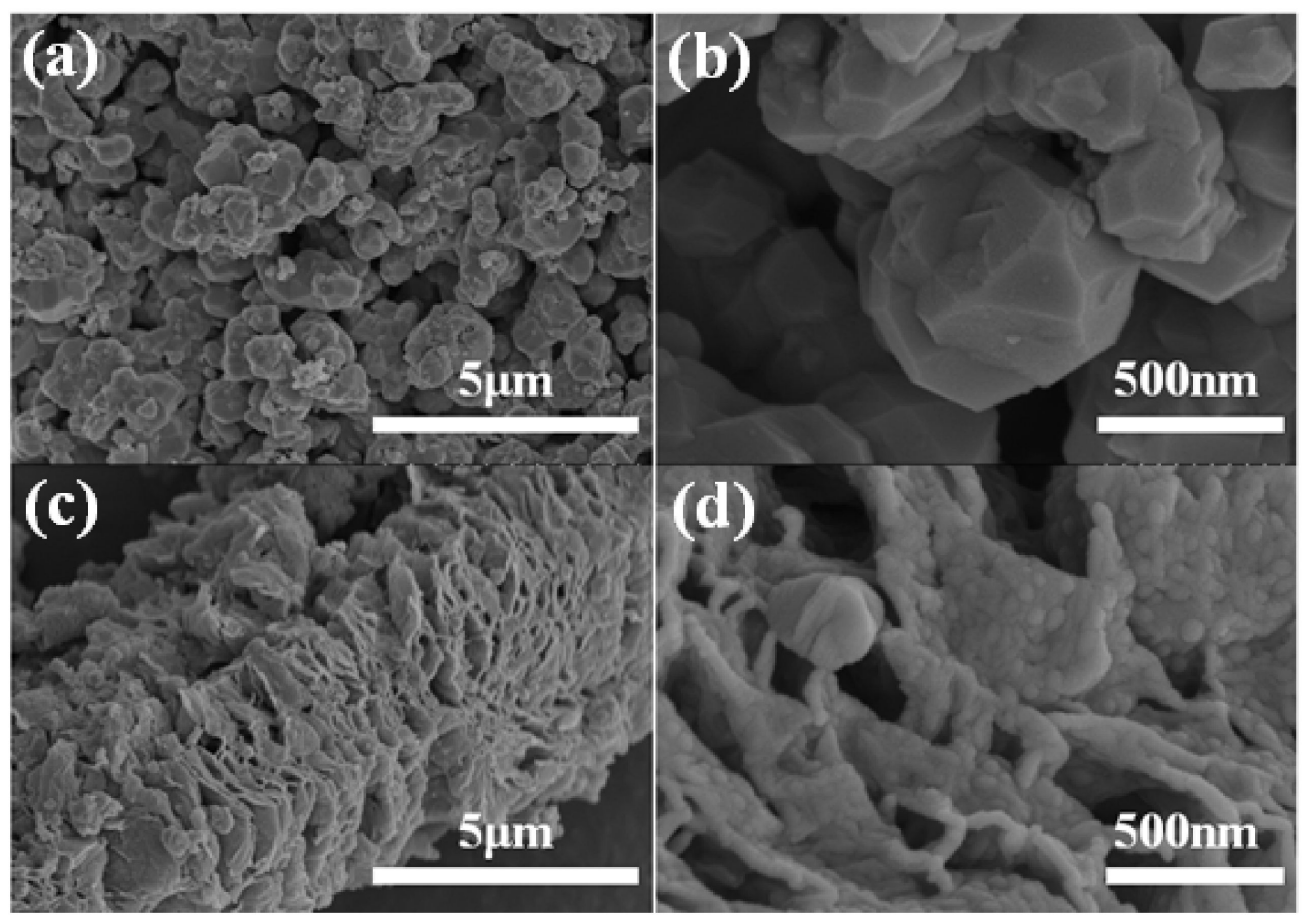
Scanning electron microscope (SEM) images of (**a**,**b**) Ge/GeO_2_ NPs, (**c**,**d**) Ge/GeO_2_/MRGO.

**Figure 4:**
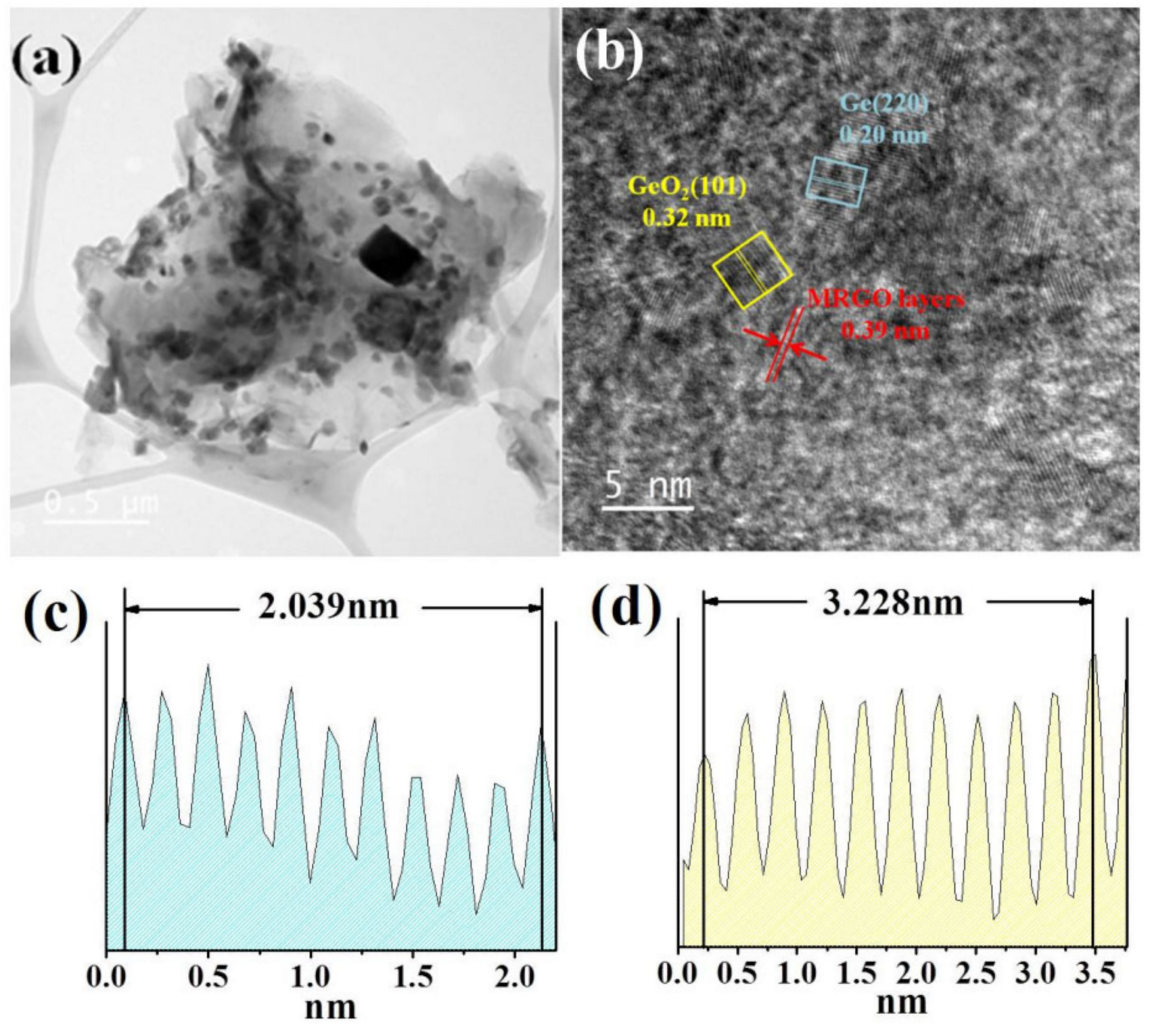
Transmission electron microscope (TEM) images of Ge/GeO_2_/MRGO in (**a**) low magnification and (**b**) high magnification. Well-deposited metallic Ge particles and the oxidized phase of Ge (GeO_2_) on the MRGO nanosheets were confirmed by the brightness profiles of (**c**) Ge (220) and (**d**) GeO_2_ (101).

Figure 3 shows the SEM images of the (a, b) Ge/GeO_2_ NPs and (c, d) Ge/GeO_2_/MRGO. As shown in Figure 3a,b, the Ge/GeO_2_ NPs agglomerated very densely, existing in the form of secondary particles. These agglomerated particles are separated into primary particles through ultrasonic treatment to form a composite with the graphene sheets, as shown in Figure 3c,d. Figure 3c,d shows the perfectly composited structure in which particles are evenly and closely embedded on the surface of the graphene sheets. In addition, for Ge/GeO_2_/MRGO, the multi-stacked graphene sheets show a bent and wrinkled shape, which is attributed to the effect of microwave treatment. On the other hand, chemically synthesized RGO showed smooth surfaces as seen in the SEM image (Figure S1 Supplementary Materials). Since the overall morphology of the samples was confirmed by SEM, HR-TEM provided more detailed information on the composite material. Figure 4 shows the TEM images of Ge/GeO_2_/MRGO. It is known that conventionally synthesized RGO consists of thin layers, as seen in the TEM image (Figure S1 in Supplementary Materials). Figure 4a shows the wide plate-shaped thin sheets with particles that were arranged in a disorderly fashion. Furthermore, at high magnification, multiple layers of the 2-dimensional material and the lattice fringe of the crystalline particles can be observed (Figure 4b). Figure 4c,d correspond to the (220) and (101) lattice planes of Ge and GeO_2_, which have d spacings of 0.20 nm and 0.32 nm, respectively. It is worth noting that the layers of the microwave-treated GO (MRGO) in the composite have an interlayer spacing of 0.39 nm. Such an increased basal spacing of MRGO in the composite could be expected to enhance ionic transport during charge/discharge reactions. Consequently, by measuring the brightness profile and calculating the interlayer distances in the image at high magnification, we confirmed that Ge/GeO_2_ NPs were homogeneously distributed onto MRGO to form the Ge/GeO_2_/MRGO composite.

**Figure 5:**
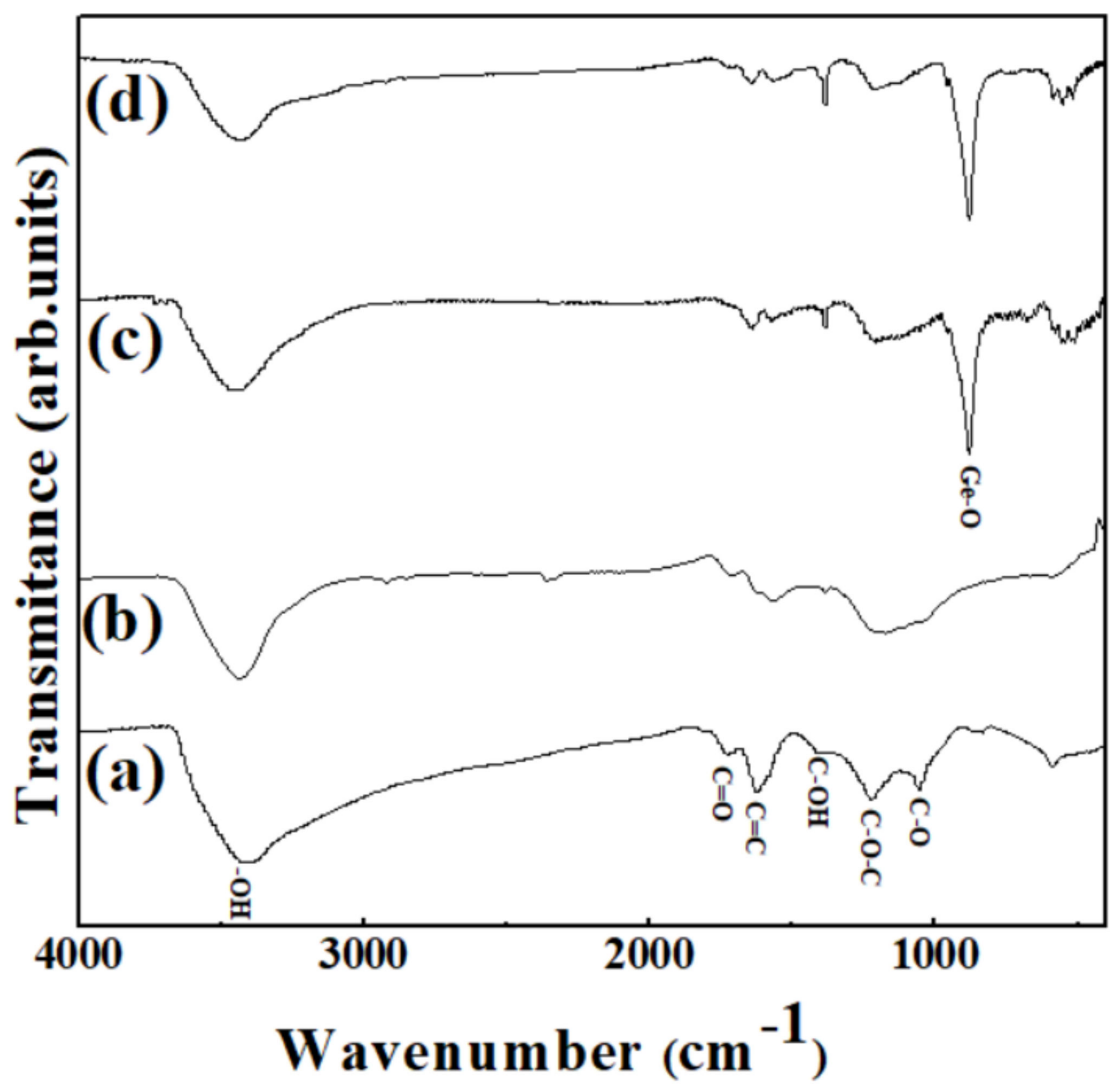
Fourier transform infrared (FT-IR) spectra of (**a**) GO, (**b**) MRGO, (**c**) Ge/GeO_2_/GO, and (**d**) Ge/GeO_2_/MRGO**.**

Figure 5 shows the FT-IR spectra of the samples. GO has various types of oxygen-containing functional groups. The broad peak at around 3406 cm^−1^ corresponds to the −OH stretching bond derived from the absorbed water (Figure 5a). The multiple bands at around 1735, 1625, 1228, and 1064 cm^−1^ are attributed to the C=O stretching frequencies of the carboxyl groups, aromatic C=C frequencies of the graphene layers, C–O symmetric stretching vibrations of the epoxy groups, and C–O stretching frequencies of the alkoxy groups, respectively. Figure 5c,d shows the spectra of the as-prepared Ge/GeO_2_/GO and the product Ge/GeO_2_/MRGO. The two distinct peaks at 880 and 554 cm^−1^ correspond to the Ge–O–Ge stretching modes and bending modes of GeO_2_, respectively [38]. Compared to GO, evidently, the relative peak intensities, derived from the oxygen-containing groups of MRGO (Figure 5b) and Ge/GeO_2_/MRGO (Figure 5d) were significantly reduced, showing that GO was successfully reduced upon the microwave treatment. Therefore, it was verified that the composite materials were properly synthesized and that a part of oxygen-containing functional groups in the product were removed sufficiently through microwave irradiation. To probe structural changes during synthesis, Raman spectra of characteristic samples (RGO and Ge/GeO_2_/MRGO) were also measured as seen in Figure S2 (Supplementary Materials). For the bare RGO, the Raman peak at about 1350 cm^−1^ was assigned to D band, while another peak at about 1595 cm^−1^ was ascribed to G band. In this Raman spectra, D band is related with elastic scattering, which was caused by vacancies, defects, and disorder of RGO. On the other hand, G band is due to the vibration of the in-plain *sp^2^* structure of graphitic framework. The relative intensity of D band to G band (I_D_/I_G_) is strongly correlated with the number of defect sites in graphitic structures and/or the domain size of graphitic *sp^2^* structures. It is well-known that the I_D_/I_G_ ratio above 1 is also an indicator whether GO was reduced to RGO. The I_D_/I_G_ ratio of Ge/GeO_2_/MRGO was similar to that of the bare RGO, clearly highlighting that the GO in the as-prepared sample was successfully reduced to RGO by microwave treatment. Furthermore, Ge/GeO_2_/MRGO showed characteristic Raman peaks of Ge and GeO_2_, indicating that microwave treatment did not deteriorate the microstructure of Ge/GeO_2_ NPs in the nanocomposite.

**Figure 6:**
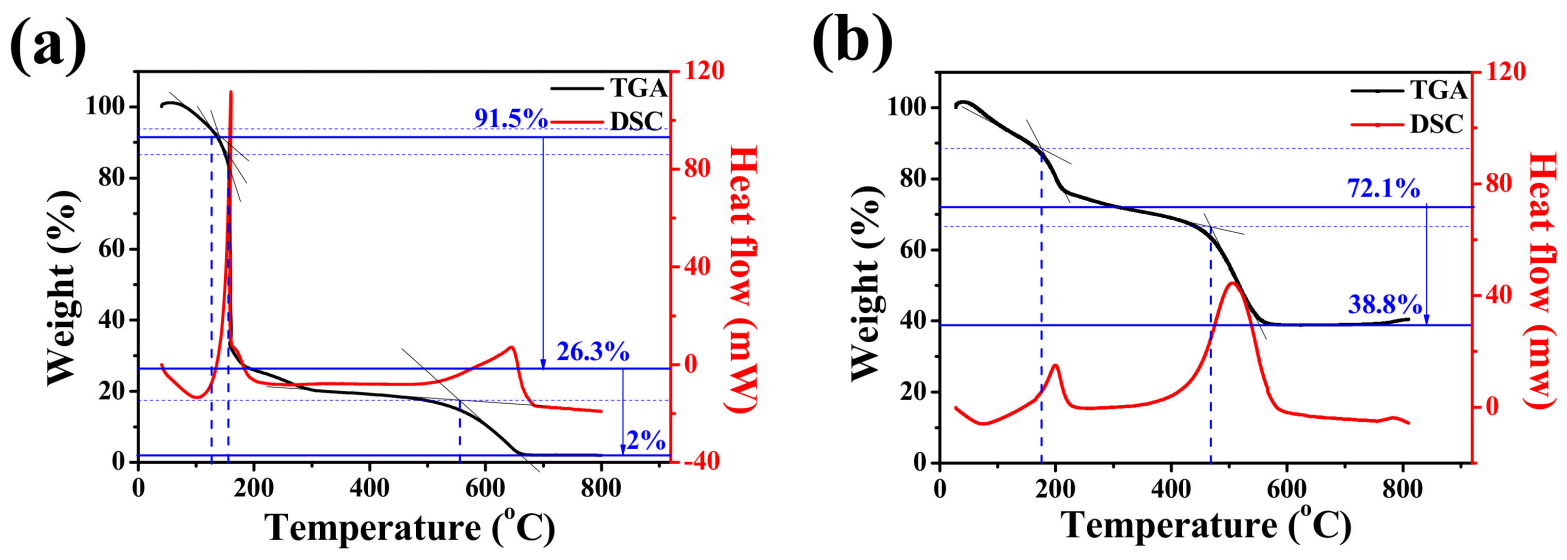
Thermogravimetric analysis (TGA) and differential scanning calorimetry (DSC) curves of (**a**) GO and (**b**) Ge/GeO_2_/MRGO.

TGA was conducted to accurately determine the weight ratio of the carbonaceous material and Ge/GeO_2_ NPs contained in the product. The TG curves of GO display three steps of weight loss processes. As shown in Figure 6a, the weight loss below 125 °C was attributed to the evaporation of the water contained in the surface of GO. The abrupt weight loss of approximately 72.8 wt%, starting at around 200 °C, was attributed to the detachment of the oxygen-containing functional groups bound to the surface of GO. Finally, the remaining carbon skeleton of the reduced GO completely burned between 400 and 700° (17.7 wt%) [39]. This enabled us to specify the weight ratio of the carbon present in the GO powder. Since the final product we synthesized would undergo microwave treatment, both the evaporation of absorbed water and the reduction of oxygen-containing groups would occur. Therefore, we must pay attention to the amount of carbonaceous materials. As shown in Figure 6b, a mass ratio of approximately 33.3 wt% was lost due to the decomposition of graphene, after which all the residual oxygen-containing functional groups disappeared before 200 °C. The amount of Ge particles is indicated by the mass that remains without burning at temperatures up to 800°, which is consistent with the experimental values of mixing GO and Ge/GeO_2_ NPs at a mass ratio of 7:3.

**Figure 7:**
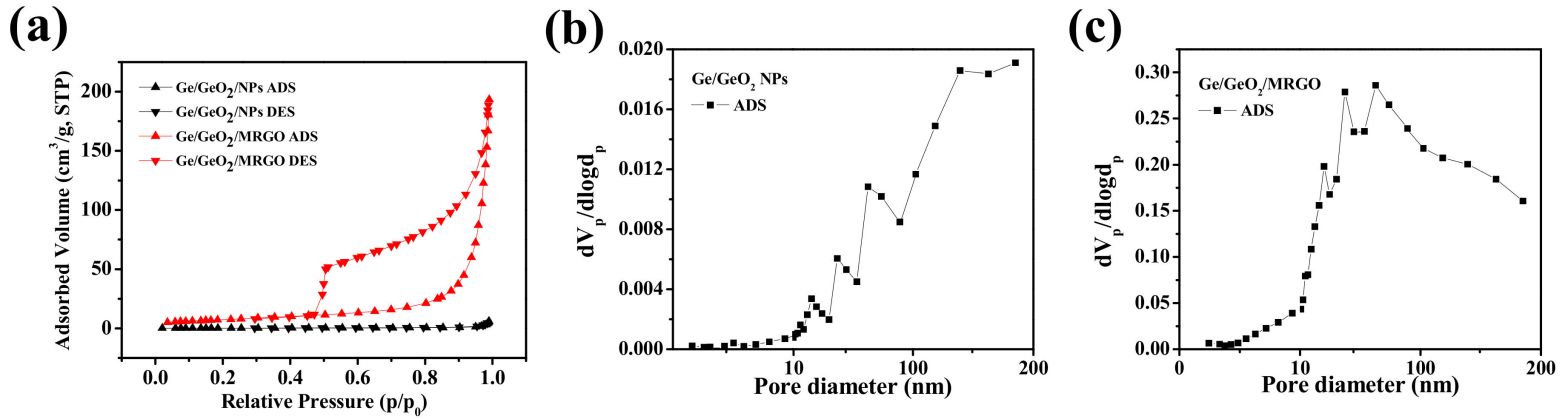
(**a**) N_2_ adsorption–desorption isotherms and the corresponding pore-size distributions of (**b**) Ge/GeO_2_ NPs and (**c**) Ge/GeO_2_/MRGO.

The porous properties of the samples were further estimated by BET surface area analysis under N_2_ gas adsorption/desorption. In Figure 7a, the bare Ge/GeO_2_ NPs exhibited a typical nonporous isotherm. Conversely, the isothermal curves of the Ge/GeO_2_/MRGO showed type-II isotherm with an H3 hysteresis loop, indicating the presence of slit-like pores and mesopores. Accordingly, the specific surface area and total pore volume increased drastically after the nanocomposites were formed compared to those of the pristine Ge/GeO_2_ NPs from 0.9 m^2^/g to 31.5 m^2^/g and 0.0092 cm^3^/g to 0.2935 cm^3^/g, respectively. Although this increase is significant, it is still much lower than that of monolayer graphene (2620 m^2^/g) [40]. This may be because of the agglomeration of randomly restacked MRGO nanosheets during the synthesis. Meanwhile, the pore-size distributions were determined using the BJH plot (Figure 7b,c). The pore size of Ge/GeO_2_/MRGO indicated the mesoporous property, and the wide distribution plot corresponded to the macroporous range. Overall, the BET/BJH and SEM/TEM results complement each other. This porous structure of Ge/GeO_2_/MRGO confers advantageous electrochemical properties and enables its application as an anode material because Li ions can easily diffuse into the structure. In addition, since it can effectively cope with the large volume change of Ge/GeO_2_ that occurs during the lithiation/delithiation process, it can provide possibilities for improving the accompanying problems, such as the fast decrease of discharge capacity and the physical pulverization of active material.

### 3.2. Electrochemical Characteristics

**Figure 8:**
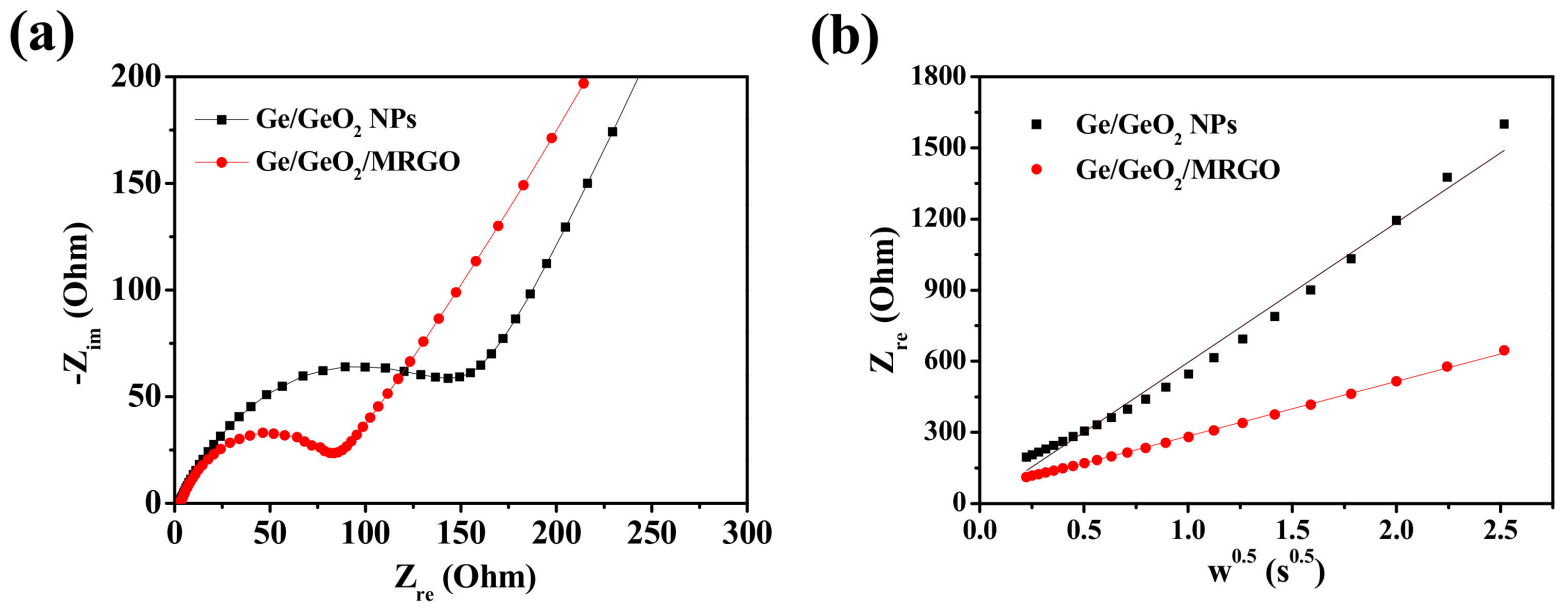
Electrochemical impedance spectroscopy (EIS) spectra of Ge/GeO_2_ NPs and Ge/GeO_2_/MRGO: (**a**) high-frequency region (Nyquist plots) and (**b**) low-frequency region (Warburg plots; plots of the real part of impedance as a function of the inverse square root of angular frequency in the Warburg region). Circles and solid lines indicate experimental and fitted spectra.

To understand the effect on compositing Ge/GeO_2_ particles with carbon nanosheets, the EIS spectra of the Ge/GeO_2_ NPs and Ge/GeO_2_/MRGO electrodes were examined. Figure 8 shows the Nyquist plots, composed of a depressed semicircle in the high-frequency region and Warburg plots, followed by a straight line at a low-frequency region. Each plot above is related to the charge-transfer resistance and Li-ion diffusion behavior. When comparing the diameter of semicircles shown in Figure 8a, compared to that for the Ge/GeO_2_ NPs, the Ge/GeO_2_/MRGO anode exhibits a much smaller semicircle. This implies that the Ge/GeO_2_/MRGO electrode has significantly higher electrical conductivity than that of the bare Ge/GeO_2_ particles. Also, the equivalent circuit model and the fitting parameters of EIS data obtained were shown in Figure S3 and Table S1 (Supplementary Materials). The equivalent circuit consists of cell components and electrolyte resistance (R_s_), surface film resistance (R_f_), charge-transfer resistance (R_ct_), constant phase element (CPE), and Warburg impedance (W). The R_f_ and the R_ct_ values of the Ge/GeO_2_/MRGO electrode (18.33 and 52.5 Ω) were much smaller than those of Ge/GeO_2_ NPs (28.9 and 101.1 Ω), clarifying the superior electrical conductivity of Ge/GeO_2_/MRGO. Additionally, the low-frequency region of EIS data is the Warburg plot (Figure 8b), in which the slope of this plot (the real part of the impedance curve in Figure 8a vs. the inverse square root of the angular frequency in the Warburg region) is an indicator of ionic conductivity [41]. As this slope decreases, the ionic conductivity increases. The observed slopes in Figure 8b clearly highlighted that the ionic conductivity of Ge/GeO_2_/MRGO was much higher than that of the bare Ge/GeO_2_ NPs. These results indicate that the introduction of Ge/GeO_2_ NPs into the matrices of MRGO not only provided more facile charge transport but also enhanced the ion conductivity through hybridization. Moreover, as expected in HR-TEM analysis, microwave treatment can lead to the large basal spacing of MRGO, which is profitable for Li-ion diffusion.

**Figure 9:**
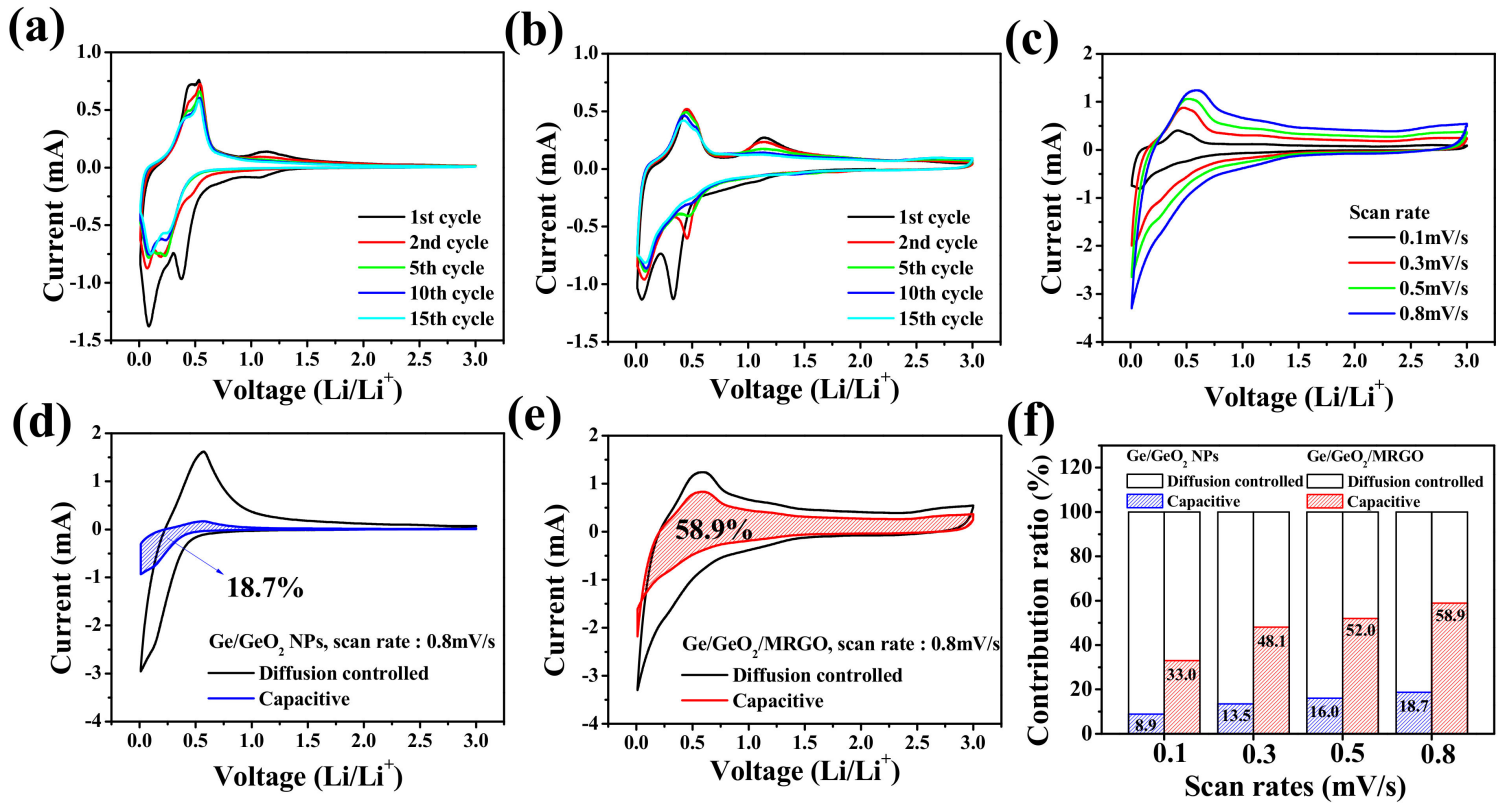
Cyclic voltammetry (CV) curves of the (**a**) Ge/GeO_2_ NPs and (**b**) Ge/GeO_2_/MRGO at a scan rate of 0.1 mV/s. (**c**) CV curves of Ge/GeO_2_/MRGO recorded at various scan rates. Comparison of pseudocapacitive and diffusion-controlled charge storage processes at scan rate 0.8 mV/s for (**d**) Ge/GeO_2_ NPs and (**e**) Ge/GeO_2_/MRGO. (**f**) Contribution ratio of pseudocapacitive and diffusion-controlled charge storage at various scan rates.

To further understand the electrochemical behavior of the samples, CV analysis was conducted. Figure 9a,b show the CV curves of the Ge/GeO_2_ NPs and the Ge/GeO_2_/MRGO electrodes in the range of 0.01–3.0 V, with a scan rate of 0.1 mV s^−1^. The two weak peaks at 1.08 V and 0.7 V, which appeared at the first cathodic scan, are indicative of the formation of amorphous Li_x_GeO_x_ and the solid-electrolyte-interphase (SEI), respectively [42,43,44,45]. The various peaks between 0.01 and 0.5 V can be attributed to the conversion reaction of GeO_x_ to Ge and the Li_x_Ge alloying process (xLi + Ge ↔ Li_x_Ge (0 ≤ x ≤ 4.4)) [46,47,48]. With the following anodic scan, a broad peak appeared from 0.4 to 0.6V, ascribed to the dealloying of Li_x_Ge, and the peak at 1.13 V indicates the reoxidation of Ge/LiO_2_ to GeO_2_ [49]. The alloying process, including the conversion reaction of the Ge/GeO_2_ NPs to amorphous Ge, can cause a peak broadening after the initial cycle [50]. While the peaks of Ge/GeO_2_ NPs, particularly those attributed to the oxidation and Li alloying, decreased rapidly, those of the germanium/graphene composite material exhibited a certain degree of maintenance. These results correspond to the fast decay of the specific capacity of Ge/GeO_2_ NPs in the early cycles and the superior capacity retention of Ge/GeO_2_/MRGO over long cycles.

In the CV test, the current measured under a potential scan rate corresponds to the kinetic information on the electrochemical behaviors during the cycle. The contribution of the total current (i) can be divided into the current associated with the diffusion-controlled process (i_diff_) and the current related to the capacitive process (i_cap_). A general description of this is the following:

i = av ^b^ = i_diff_ + i_cap_ (3)

log i = log a + b log v(4)

In this power-law relationship, both a and b are the adjacent parameters. Herein, parameter b corresponds to the slope in the linear equation above (Equation (4)) and has a value between 0.5 and 1. The typical battery-type anode materials, which are highly influenced by the diffusion-controlled process, have a b value close to 0.5, and the b value of the pseudocapacitive electrode is close to 1 [51,52,53,54]. Figure 9c shows the CV curves of Ge/GeO_2_/MRGO at different scan rates (0.1, 0.3, 0.5, and 0.8 mV/s), and the dependence of the b value, which was obtained from the cathodic and anodic sweeps, are shown in Figure S4 (Supplementary Materials). For Ge/GeO_2_/MRGO, b values of 0.72 and 0.85, relatively close to 1 on average, were determined for the cathodic and anodic scan, respectively, indicating that the contribution of the surface-controlled process to the Li-ion storage mechanism was more dominant than that of the diffusion-controlled storage mechanism. A more quantitative comparison of the contribution of the diffusion-control and capacitive processes is shown in Figure 9d–f. For the Ge/GeO_2_ NP electrode containing Ge, which is well known to follow the alloying–dealloying mechanism and GeO_2_ conversion reaction, it has been demonstrated that there is little influence by a capacitance of ~18.7%, at a scan rate of 0.8 mV/s. Contrarily, for the nanocomposite to which the conductive MRGO was incorporated, it could be observed that the expressed capacity contributed by the pseudocapacitive contribution increased significantly to 58.8% at the same scan speed.

**Figure 10:**
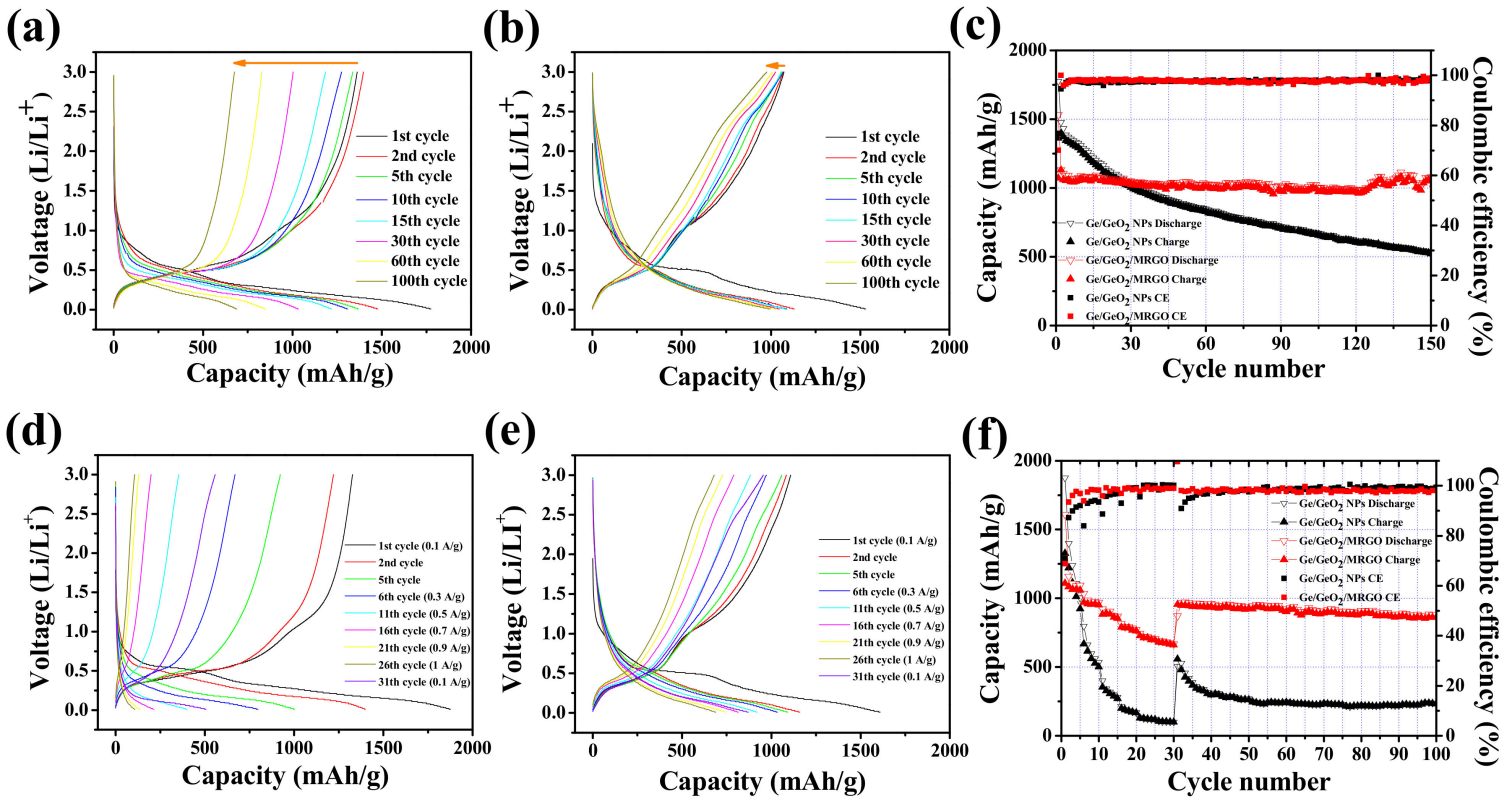
Charge/discharge curves of (**a**) Ge/GeO_2_ NPs and (**b**) Ge/GeO_2_/MRGO (at a current density of 100 mA/g), and (**c**) their cycling performances. Charge/discharge curves of (**d**) Ge/GeO_2_ NPs and (**e**) Ge/GeO_2_/MRGO at current densities of 100, 300, 500, 700, 900, and 1000 mA/g for 5 successive cycles (after 30 cycles the current density was reverted back to 100 mA/g), and (**f**) their cycling performance and rate capability.

Figure 10a,b shows the galvanostatic charge/discharge curves of the Ge/GeO_2_ NPs and Ge/GeO_2_/MRGO versus Li at a current density of 100 mAh/g between 0.01 and 3 V. The initial discharge/charge capacities of the Ge/GeO_2_ NPs and Ge/GeO_2_/MRGO were obtained to be 1775/1368 mAh/g and 1531/1075 mAh/g, corresponding to the initial coulombic efficiencies (ICEs) of 76.7% and 70.2%, as shown in Figure 10c, respectively. These high first discharge capacities, exceeding the theoretical values, were attributed to the irreversible formation of SEI layers on the surface of the electrodes. From the second cycle, the opposite trend from the beginning was observed, indicating the CEs of the samples as 97.2% and 98% (average values up to the 30th cycle). This confirms that Ge/GeO_2_ NPs cannot effectively withstand the pulverization of the electrode caused by the repeated lithiation/delithiation, resulting in the severe capacity fading during the initial few cycles. The cracks that were generated by the destruction of the electrode could continuously expose new active surfaces, and Li ions are additionally consumed for the SEI film formation. In contrast, well deposited Ge/GeO_2_ NPs on the graphene networks of Ge/GeO_2_/MRGO can effectively minimize the decrease in CE, resulting in good cycling performance. For the prolonged cycles, Ge/GeO_2_/MRGO showed stable cyclability over 150 cycles (discharge capacity of 1080 mAh/g at the 150th cycle), while Ge/GeO_2_ NPs only reported 30% capacity retention (discharge capacity of 533 mAh/g at the 150th cycle). The alloying–dealloying processes are represented in the voltage profiles as plateaus observed at around 0.5 V for the charge curves and 0.1 V for the discharge curves, obtained over long-term cycles for both samples; this suggests a high level of reversibility. However, the discharge plateaus of 0.45 V and charge plateaus of 1.1 V, attributable to the reduction of GeO_2_ into Ge and Li_2_O and the reoxidation of elemental Ge, were diminished in the Ge/GeO_2_ NP electrode before and after the fifth cycle. This is mainly because of the irreversibility of the conversion reaction by the lithium oxides generated during the discharge processes. By contrast with the Ge/GeO_2_ NPs, Ge/GeO_2_/MRGO showed better capacity retention to restore the reversibility of the GeO_2_ conversion by introducing conductive MRGO networks to the Ge/GeO_2_ particles. The rate capabilities and cycling performance were evaluated at various current densities of 100, 300, 500, 700, 900, 1000 mA/g and returning to 100 mA/g (Figure 10d–f). As the current density increased, the Ge/GeO_2_ NPs and Ge/GeO_2_/MRGO exhibited specific capacities of 1325, 631, 328, 186, 124, and 103 mAh/g and 1212, 986, 893, 791, 720, and 680 mAh/g, respectively (average capacities of all five cycles at each current density). At the 50th cycle, after the current density recovered to 100 mA/g, Ge/GeO_2_/MRGO showed a capacity of 940 mAh/g with a good retention rate (77%) similar to that during the initial five cycles, while the bare Ge/GeO_2_ NPs showed poor cyclability, and their specific capacity decreased to 269 mAh/g (only 20% capacity retention). To the best of our knowledge, the discharge capacity of Ge/GeO_2_/MRGO is one of the excellent values reported so far for Ge-containing electrodes (Table S2 in Supplementary Materials). It is worth noting that, the present Ge/GeO_2_/MRGO showed very high discharge capacity even though a wide potential window (from 0.01 to 3 V) was applied during charge/discharge cycles. From the above findings, it can be concluded that the MRGO networks afford excellent electrical conductivity and a short ion diffusion pathway to Ge/GeO_2_/MRGO, enabling improved Li insertion/extraction processes.

## 4. Conclusions

The nanocomposite consisting of Ge/GeO_2_ NPs and reduced GO were successfully prepared via the reaction between each colloids and the subsequent microwave-assisted syntheses. The structural features of the prepared sample were characterized well by XRD and XAS. Compared to those of the bare Ge/GeO_2_ NPs, Ge/GeO_2_/MRGO, with a conductive carbon network added, exhibited a higher porosity and specific surface area (BET), and through EIS analysis we verified the enhanced ionic conductivity and charge transfer, which are important factors for Li-ion storage. In the charge/discharge tests, the model compound was evaluated to exhibit excellent reversible capacity and rate capability, and a more detailed discussion of the ion storage mechanism via CV analyses was provided.

## Data Availability

The data presented in this study are available on request from the corresponding author.

## Supplementary Materials

The following are available online at https://www.mdpi.com/2079-4991/11/2/319/s1, Figure S1: SEM and TEM images of RGO, Figure S2: Raman spectra of RGO and Ge/GeO2/MRGO, Figure S3: The equivalent circuit model for the EIS data fitting, Figure S4: A power-law relationship of Ge/GeO2 NPs and Ge/GeO2/MRGO, Table S1: Parameters derived by using equivalent circuit model for the EIS data of Ge/GeO2 NPs and Ge/GeO2/MRGO electrodes, Table S2: Summary of recent works for the various Ge and GeO2 composites anode materials used in lithium ion batteries.
