# Supplementary material for: Microwave-Assisted Synthesis of Ge/GeO2-Reduced Graphene Oxide Nanocomposite with Enhanced Discharge Capacity for Lithium-Ion Batteries
Source: Nanomaterials (Basel). 2021 Jan 27;11(2):319. doi: 10.3390/nano11020319 (PMC7911565; doi:10.3390/nano11020319)
Supplement: Supplementary file 1 [file nanomaterials-11-00319-s001.pdf]

Supplementary material

Article

Microwave-Assisted Synthesis of Ge/GeO_2-Reduced Graphene Oxide Nanocomposite with Enhanced Discharge Capacity for Lithium-Ion Batteries

Ji-Hye Koo and Seung-Min Paek *

Department of Chemistry, Kyungpook National University, Daegu 41566, Korea; guzza95@naver.com
* Correspondence: smpaek@knu.ac.kr; Tel.: +82-53-950-5335

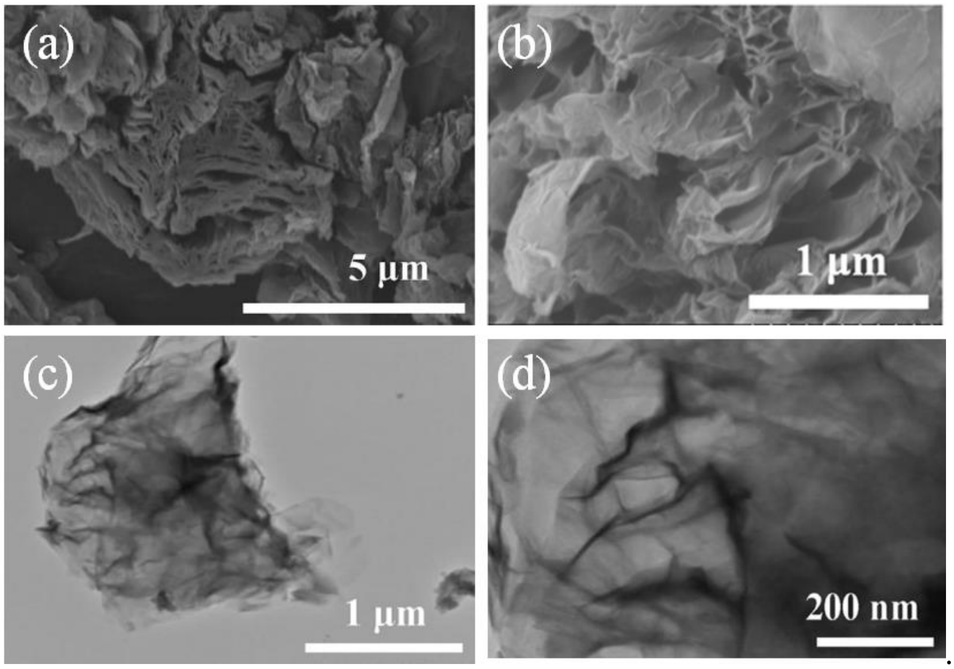

Figure S1. (a,b) SEM images (c,d) TEM images of RGO.

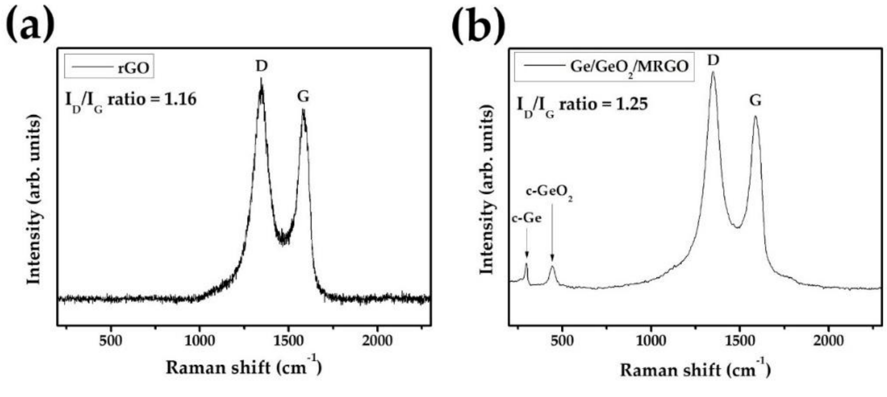

Figure S2. Raman spectra of (a) RGO and (b) Ge/GeO_2/MRGO.

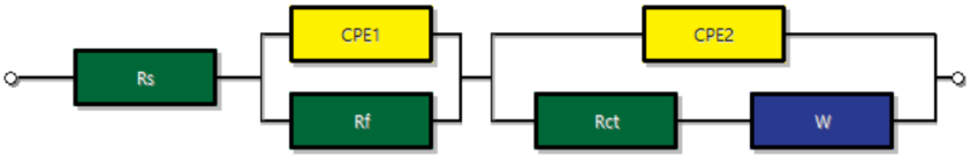

Figure S3. The equivalent circuit model for the EIS data fitting.

Table S1. Parameters derived by using equivalent circuit model for the EIS data of Ge/GeO_2 NPs and Ge/GeO_2/MRGO electrodes: R_s (bulk resistance), R_f (film resistance), and R_{ct} (charge-transfer resistance).

**Anode materials**	**R_s (Ω)**	**R_f (Ω)**	**R_{ct} (Ω)**
Ge/GeO_2 NPs	3.7	28.9	101.1
**Ge/GeO_2/MRGO.**	3.5	18.3	52.5

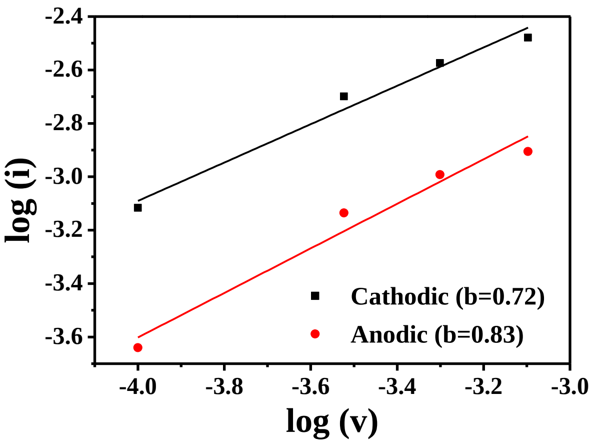

Figure S4. A power-law relationship of Ge/GeO_2 NPs and Ge/GeO_2/MRGO

Table S2. Summary of recent works for the various Ge and GeO_2 composites anode materials used in lithium ion batteries; [a]capacity (mA h/g); [b]current density (mA/g); [c]mass ratio (active material : conductive carbon : binder).

Anode materials	Capacity (Capacity[a]/Current density[b]/Cycle number)	Voltage range (vs. Li/Li^+)	Mass ratio[c]	Ref.
Ge NWs in graphite tubes	260/250/100	0.001–2.5 V	7 : 2 : 1	[1]
Porous Ge microtubes	1200/138/100	0.01–1.5 V	8 : 1 : 1	[2]
3D porous Ge NPs	1420/1600/200	0.01–1.5 V	8 : 1 : 1	[2]
Ge–graphene–carbon nanotube	864/100/100	0.01–3.0 V	8 : 1 : 1	[3]
Ge/RGO-1	815/200/100	0.01–1.5 V	8 : 1 : 1	[4]
Ge/RGO-2	960/200/100	0.01–1.5 V	8 : 1 : 1	[4]
Ge/RGO-3	720/200/100	0.01–1.5 V	8 : 1 : 1	[4]
GeO_2/graphene	650/100/80	0.0–3.0 V	8 : 1 : 1	[5]
Ge/GeO_2/MRGO	1080/100/150	0.01–3.0 V	7 : 2 : 1	This work

References

1. Sun, Y.; Jin, S.; Yang, G.; Wang, J.; Wang C. Germanium Nanowires-in-Graphite Tubes via Self-Catalyzed Synergetic Confined Growth and Shell-Splitting Enhanced Li-Storage Performance. *ACS Nano,* **2015**, *9, 4,* 3479–3490.

2. Li, D.; Feng, C.; Liu, H.K.; Guo, Z. Hollow carbon spheres with encapsulated germanium as an anode material for lithium ion batteries. *J. Mater. Chem. A*, **2015**, *3*, 978.

3. Li, X.; Guo, W.; Wana Q.; Ma J. Porous amorphous Ge/C composites with excellent electrochemical properties. *RSC Adv*, **2015**, *5*, 28111.

4. Chen, Y.; Ma, L.; Shen, X.; Ji, Z.; Yuan, A.; Xu, K.; Shah, S.A. In-situ synthesis of Ge/reduced graphene oxide composites as ultrahigh rate anode for lithium-ion battery. *Journal of Alloys and Compounds*, **2019**, *801*, 90–98.

5. Wei, W.; Tian, A.; Jia, F.; Wang, K.; Qu, P.; Xu, M. Green synthesis of GeO_2/graphene composites as anode material for lithium-ion batteries with high capacity. *RSC Adv*, **2016**, *6*, 87440.
